# *Hermetia illucens* in diets for zebrafish (*Danio rerio*): A study of bacterial diversity by using PCR-DGGE and metagenomic sequencing

**DOI:** 10.1371/journal.pone.0225956

**Published:** 2019-12-10

**Authors:** Andrea Osimani, Vesna Milanović, Andrea Roncolini, Paola Riolo, Sara Ruschioni, Nunzio Isidoro, Nino Loreto, Elena Franciosi, Kieran Tuohy, Ike Olivotto, Matteo Zarantoniello, Federica Cardinali, Cristiana Garofalo, Lucia Aquilanti, Francesca Clementi

**Affiliations:** 1 Dipartimento di Scienze Agrarie, Alimentari ed Ambientali, Università Politecnica delle Marche, Ancona, Italy; 2 Food Quality and Nutrition Department (DQAN), Research and Innovation Center, Fondazione Edmund Mach (FEM), San Michele all’Adige, Italy; 3 Dipartimento di Scienze della Vita e dell'Ambiente, Università Politecnica delle Marche, Ancona, Italy; Universita degli Studi della Basilicata, ITALY

## Abstract

In the present research, bacterial diversity was studied during a 6-month feeding trial utilizing zebrafish (*Danio rerio*) fed *Hermetia illucens* reared on different substrates with an emphasis on fish gut bacterial diversity. A polyphasic approach based on viable counting, PCR-DGGE and metagenomic 16S rRNA gene amplicon target sequencing was applied. Two different *H*. *illucens* groups were reared on coffee by-products (C) or a mixture of vegetables (S). Viable counts showed a wide variability based on substrate. PCR-DGGE and Illumina sequencing allowed the major and minor bacterial taxa to be detected. Both samples of larvae and their frass reared on the S substrate showed the highest richness and evenness of bacterial communities, whereas zebrafish (ZHC) fed *H*. *illucens* reared on substrate C and zebrafish (ZHS) fed *H*. *illucens* reared on substrate S had the lowest bacterial richness and evenness. A stimulating effect of bioactive compounds from coffee by-products on the occurrence of Lactobacillaceae and Leuconostoccaceae in *H*. *illucens* reared on substrate C has been hypothesized. Zebrafish gut samples originating from the two feeding trials showed complex microbial patterns in which Actinobacteria and Alteromonadales were always detected, irrespective of the diet used. Enterobacteriaceae in fish guts were more abundant in ZHS than in ZHC, thus suggesting an influence of the bioactive compounds (chlorogenic and caffeic acids) in the substrate on Enterobacteriaceae in fish guts. ZHC showed a higher abundance of Clostridia than did ZHS, which was likely explained by stimulating activity on the bacteria in this class by the bioactive compounds contained in *H*. *illucens* reared on substrate C. An influence of the microbiota of *H*. *illucens* or insect-derived bioactive compounds on the gut microbiota of zebrafish has been suggested. The presence of bacteria consistently associated with zebrafish guts has been found irrespective of the diet, thus attesting to the likely stability of the core fish microbiota.

## Introduction

The use of edible insects as food and feed could represent a suitable source of alternative protein due to their high-quality nutritional values and the environmental sustainability of their rearing [1, 2]. Indeed, edible insects can grow on substrates deriving from food industry by-products, thus adding value to these organic matrices. Moreover, edible insects are already reared in Asia, Africa, Oceania and South America, thus representing a growing source of income [3, 4]. In the European Union (EU), stakeholders have recently begun paying attention to the applications of edible insects as food or feed. In 2015, the European Food Safety Authority (EFSA) listed a few insect species with potential use as food and feed in the EU. The EFSA list included the following species: *Musca domestica*, *Hermetia illucens*, *Tenebrio molitor*, *Zophobas atratus*, *Alphitobius diaperinus*, *Galleria mellonella*, *Achroia grisella*, *Bombyx mori*, *Acheta domesticus*, *Gryllodes sigillatus*, *Locusta migratoria migratorioides*, and *Schistocerca americana* [5]. The EFSA also highlighted the need for in-depth studies to assess the safety of edible insects to achieve large-scale production [6]. Since insects can be natural reservoirs of microorganisms that can be transferred to the environment and to living animals used as food sources, their microbiological assessment must be properly conducted [7].

More recently, the European Commission issued Regulation No. 2017/893 of 24 May 2017 that allowed the use of insects as processed animal proteins (PAPs) in aquaculture [8]; such a Regulation paved the way for a more concrete exploitation of edible insects in the EU. In such a context, *H*. *illucens* (Diptera:Stratiomyidae) (commonly known as black soldier fly) represents one of the most promising species to be used in aquaculture [4, 9–13]. Indeed, *H*. *illucens* is economically among the most important farmed insects in the world [14]. It is noteworthy that, although many studies have already been carried out to optimize the rearing conditions and to increase the yield of the bioconversion process, data on microbiological aspects of this insect are still scarce [15]. Recent studies have highlighted that *H*. *illucens* larvae can be carriers of microorganisms whose presence may be influenced in part by the rearing substrate. De Smet et al. [16] recently reviewed the composition of microbial communities of *H*. *illucens* larvae reared on different substrates, such as food waste, cooked rice, calf forage and the so-called Gainesville housefly diet. The authors reported that the presence of a core microbiota in the insect gut can be conceivable, although the different rearing substrates affected the relative abundances of the detected microbial taxa [16]. Similarly, Wynants et al. [17], highlighted that *H*. *illucens* larvae reared at several locations showed the presence of a core microbiota among which the phyla Firmicutes and Proteobacteria were dominant. The occurrence of interacting factors, both abiotic and biotic, capable of affecting the larval microbiota, was also considered by the same authors [17]. Interestingly, Bruno et al. [15] reported a variable influence of ingested food on the microbiota of different gut regions of *H*. *illucens* larvae. The same study reported that an excessively high protein content in the feeding substrate seemed to favor midgut dysbiosis. Furthermore, an influence of the rearing substrate on *H*. *illucens* larval mycobiota was also highlighted by [18].

In the present research bacterial diversity was studied during a 6-month feeding trial with zebrafish (*Danio rerio*) fed *H*. *illucens* reared on different substrates, with emphasis on fish gut bacterial diversity. To this end, a polyphasic approach based on viable counting, Polymerase Chain Reaction-Denaturing Gradient Gel Electrophoresis (PCR-DGGE) and metagenomic 16S rRNA gene amplicon target sequencing (Illumina sequencing) was applied.

## Materials and methods

### Insect rearing

A *Hermetia illucens* laboratory colony was established from commercially available larvae (Smart Bugs, Ponzano Veneto, Italy). The rearing was maintained under controlled conditions (27°C ±2°C, 70% ±10% RH, and 14:10 light:dark photoperiod). Larvae were placed in separate sterile plastic boxes (57 × 39 × 28 cm) until pupal stage was reached. Newly emerged adults were placed in two different mating and oviposition cages (2 m^3^). After two days, coffee by-products (C) (coffee silverskin obtained from the roasting process at Saccaria Caffè SRL, Marina di Montemarciano, Italy) or vegetable substrate (S) composed of 50% corn meal and fruit and 50% vegetable mixture were provided, in small sterile plastic boxes (16.5 × 22 cm), as egg-laying substrates. The plastic boxes were changed every 2 days and the eggs collected using brushes. Four thousand 4-day-old larvae (individually counted using tweezers and brushes) were transferred in sterile plastic boxes (57 × 39 × 28 cm) and fed with C (namely, HC larvae) or S (namely, HS larvae). The feeding substrate was supplied *ad libitum* to the developing larvae. The experiments were performed in triplicate for each rearing condition. Larval rearing boxes were covered with a lid provided with a single ventilation hole and equipped with a ramp (35°) and a collection device. When the larvae reached the prepupal stage, they left the feeding substrate and climbed up the ramp into the collecting vessel. Both prepupal groups (HC and HS) were collected, freeze-dried, and processed to obtain full-fat prepupae fish meals. Moreover, HC, HS, C, S and frass samples, composed of excrement from larvae mixed with substrate residues and exuviae, (namely, HCF from HC or HSF from HS) were analyzed as described below.

### Zebrafish rearing

*Danio rerio* fed with either 100% HC or 100% HS were produced as already reported by Zarantoniello et al. [13]. *D*. *rerio* were spawned and maintained under the rearing conditions already reported by Zarantoniello et al. [13]. Embryos were then collected, counted under a stereomicroscope (Leica Wild M3B, Leica Microsystems, Nussloch, Germany) and randomly divided into two experimental groups (in triplicate) according to the two test diets [13]. Fish (1500 larvae per dietary treatment; 3 × 20 L tanks containing 500 larvae each) were daily fed (2% body weight) the two different insect meals (HC; HS) for 6 months according to Vargas et al. [12] and Zarantoniello et al. [19]. At the end of the experimental period fish were anesthetized with a lethal dose of MS222 (1 g/L, Sigma Aldrich, Saint Louis, Missouri, USA). All efforts were made to minimize animal suffering [16]. The microbiota of ZHC (gut contents of zebrafish fed HC) and ZHS (gut contents of zebrafish fed HS) were then analyzed.

### Microbiological analyses

Microbiological enumeration was carried out on samples C, HC, HCF, S, HS and HSF. Ten grams of each sample were aseptically diluted in 90 mL of sterile peptone water (bacteriological peptone 1 g/L) and homogenized in a Stomacher 400 Circulator apparatus (VWR International PBI, Milan, Italy). Homogenate ten-fold dilutions were inoculated on specific solid media for the enumeration of the following microorganisms: total mesophilic aerobes counted according to the UNI EN ISO 4833:2004 standard method; Enterobacteriaceae counted in according to the ISO 21528–2:2004 standard method; and bacterial spores, for which homogenized samples were subjected to thermal treatment at 80°C for 15 min followed by cooling in iced water to inactivate the vegetative cells [20] and then grown in Standard Plate Count Agar (Oxoid, Basingstoke, UK) incubated at 30°C for 48 h. Presumptive lactic acid bacteria counts were carried out on MRS agar (Oxoid) with cycloheximide (250 mg/L) added, incubated at 37°C for 72 h under anaerobiosis.

The presence of *Listeria monocytogenes* and *Salmonella* spp. was assessed in accordance with AFNOR BIO 12/11-03/04 and AFNOR BIO 12/16-09/05 standard methods, respectively.

Analyses were carried out in duplicate. The results were subjected to ANOVA using the software JMP v.11.0.0 (SAS Institute Inc., Cary, NC) and expressed as the mean of log colony forming units (cfu) per gram ± standard deviation of independent duplicate biological experiments.

### Molecular analyses

To gain insight into the microbial species occurring in the analyzed samples, C, HC, HCF, ZHC, S, HS, HSF and ZHS were subjected to culture-independent analyses via PCR-DGGE and Illumina sequencing. To this aim, DNA extraction from samples was carried out as already described by Garofalo et al. [21]. DNA extracts were standardized to 25 ng/μL, and equal portions derived from the biological replicates of each sample were pooled together and vortexed vigorously.

PCR DGGE analysis was performed as described by Milanović et al. [22] through PCR amplification of the V3 region of the 16S rRNA gene using universal prokaryotic primers 338f (5’-ACT CCT ACG GGA GGC AGC AGC AG-3’) added with GC clamp and 518r (5’-ATT ACC GCG GCT GCT GG-3’) [23]. After the DGGE, bands were excised and reamplified and amplicons sent for sequencing to Genewiz (Leipzig, Germany). The output sequences in FASTA format were compared with those deposited in the GenBank DNA database using the basic BLAST search tools [24].

Samples were further subjected to Illumina analysis. In more detail, a 464-nucleotide sequence of the bacterial V3-V4 region [25, 26] of the 16S rRNA gene (*Escherichia coli* positions 341 to 805) was amplified and processed as already described by Osimani et al. [27].

Unique barcodes were attached before the forward primers; the amplicons were cleaned using the Agencourt AMPure kit (Beckman coulter). The DNA concentration of amplicons was determined using the Quant-iT PicoGreen dsDNA kit (Invitrogen). The quality of the generated amplicon libraries was evaluated with a Bioanalyzer 2100 (Agilent, Palo Alto, CA, USA) using the High Sensitivity DNA Kit (Agilent). Raw paired-end FASTQ files were demultiplexed using idemp (https://github.com/yhwu/idemp/blob/master/idemp.cpp) and imported into Quantitative Insights Into Microbial Ecology (Qiime2, version 2018.2) [28]. Sequences were quality filtered, trimmed, denoised, and merged using DADA2 [29]. Chimeric sequences were identified and removed via the consensus method in DADA2. Representative sequences were aligned with MAFFT and used for phylogenetic reconstruction in FastTree using plugins alignment and phylogeny [30, 31]. Alpha and beta diversity metrics were calculated using the core-diversity plugin within QIIME2 and emperor [32]. Taxonomic and compositional analyses were conducted with plugins feature-classifier (https://github.com/qiime2/q2-feature-classifier). A pretrained Naive Bayes classifier based on the Greengenes 13_8 97% operational taxonomic units (OTUs) database (http://greengenes.secondgenome.com/), which had been previously trimmed to the V4 region of 16S rDNA, bound by the 341F/805R primer pair, was applied to paired-end sequence reads to generate taxonomy tables.

The data generated by Illumina sequencing were deposited in the NCBI Sequence Read Archive (SRA) and are available under Ac. PRJNA546499.

## Results and discussion

*H*. *illucens* larvae are able to efficiently convert organic by-products into biomass to be used for feed or other purposes. Hence, *H*. *illucens* larvae constitute a potentially suitable ingredient for fish feed, as well as a good marketing opportunity for overcoming concerns regarding the use of fishmeal in aquaculture [4, 11]. Notwithstanding, studies on microbiological aspects of the use of *H*. *illucens* as feed are surprisingly very limited [15].

As reported in Table 1, except for HCF, the samples derived from coffee by-products (C and HC) were characterized by high counts for all the microbial groups assessed. Of note, Enterobacteriaceae counts in samples HC and HCF were lower than that in sample C, reflecting the previously described inhibitory effect of *H*. *illucens* larvae on Enterobacteriaceae [33]. Surprisingly, the inhibitory effect was not observed in HS or HSF, which exhibited higher Enterobacteriaceae loads than in S samples. The data collected on mesophilic aerobes, bacterial spores, and LAB were consistent with those reported by Wynants et al. [17] that reflected a wide variability based on the substrate, the rearing methods and the timing of larval harvest. In frass samples, viable counts were generally higher, probably because of the accumulation effect during the rearing period [27]. Finally, no *Listeria monocytogenes* or *Salmonella* spp. were detected in any of the analyzed samples. Raw data used for statistical analysis of viable counts are reported in S1 Table.

**Table 1:** Viable counts from the analyzed samples.

Samples	Total mesophilic aerobes	Bacterial spores	Lactic acid bacteria	Enterobacteriaceae
Rearing chain 1				
C	8.13±0.03 ^a^	7.45±0.02 ^b^	7.42±0.01 ^a^	5.35±0.06 ^a^
HC	6.08±0.00 ^c^	5.92±0.01 ^c^	3.89±0.10 ^c^	3.30±0.02 ^b^
HCF	7.77±0.03 ^b^	7.71±0.04 ^a^	6.29±0.08 ^b^	<1 ^c^
Rearing chain 2				
S	5.83±0.04 ^c^	2.53±0.04 ^c^	3.93±0.04 ^c^	4.18±0.08 ^c^
HS	7.13±0.03 ^b^	3.36±0.08 ^b^	7.00±0.00 ^b^	6.90±0.01 ^b^
HSF	10.50±0.06 ^a^	4.14±0.01 ^a^	7.46±0.01 ^a^	7.48±0.01 ^a^

Results are expressed as log colony forming units (cfu) g^-1^. For microbial counts, means ± standard deviations of triplicate independent experiments are shown. For each rearing chain, within each column, means with different superscript letters are significantly different (*P* < 0.05). C, coffee by-product; HC, *H*. *illucens* reared on coffee by-product; HCF, frass of *H*. *illucens* reared on coffee by-product; S, vegetable substrate; HS, *H*. *illucens* reared on vegetable substrate; HSF, frass of *H*. *illucens* reared on vegetable substrate.

The data on the overall microbiota collected highlighted very complex populations in almost all the samples where, thanks to the combination of PCR-DGGE and Illumina sequencing, major and minor taxa were detected.

**Fig 1:**
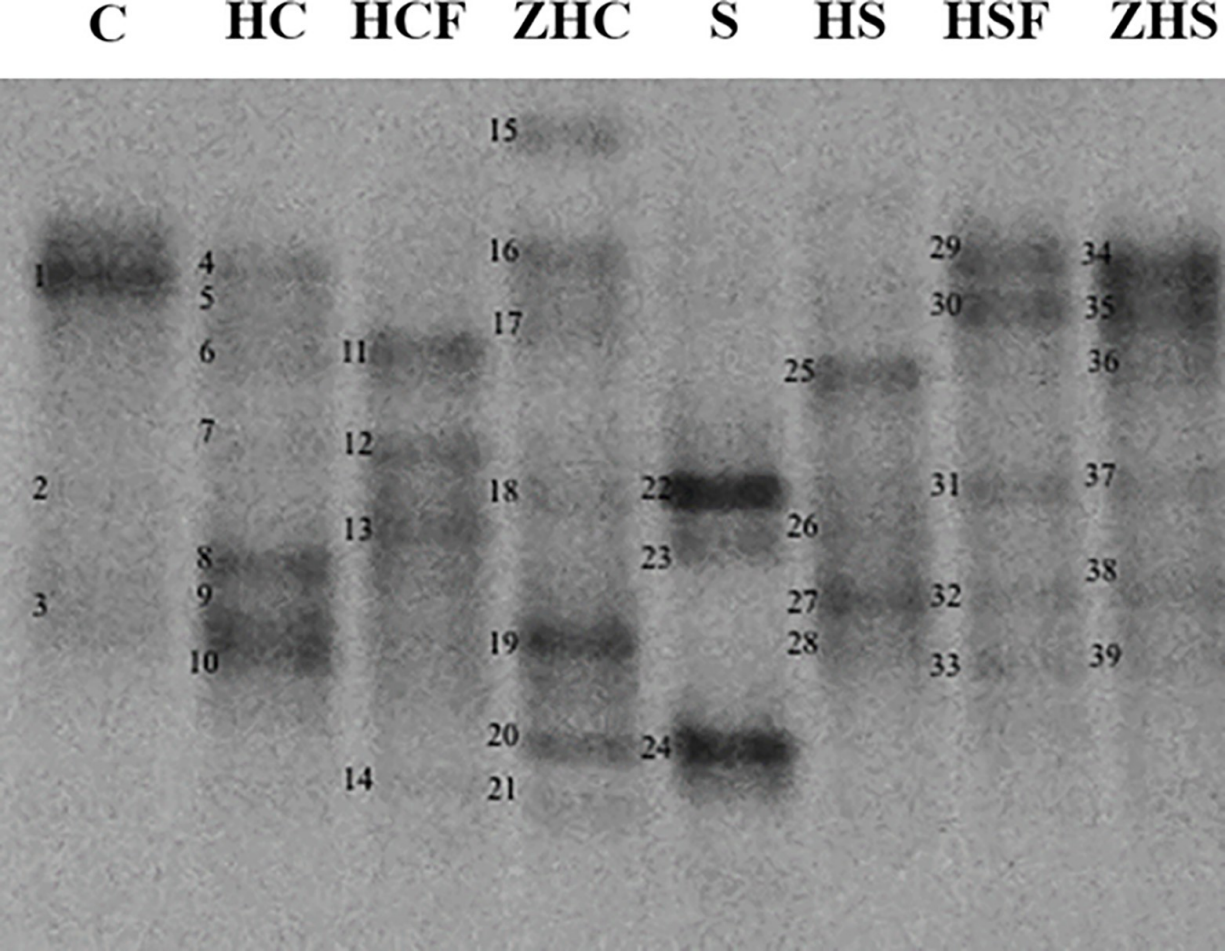
Bacterial DGGE profiles of the DNA extracted directly from the analyzed samples and amplified with primers 338fGC and 518r. The bands indicated by the letters were excised, reamplified and subjected to sequencing. The identities of the bands are given in Table 2. C, coffee by-product; HC, *H*. *illucens* reared on coffee by-product; HCF, frass of *H*. *illucens* reared on coffee by-product; S, vegetable substrate; HS, *H*. *illucens* reared on vegetable substrate; HSF, frass of *H*. *illucens* reared on vegetable substrate; ZHC, gut content of zebrafish fed HC; ZHS, gut content of zebrafish fed HS.

Regarding PCR-DGGE analysis, the closest relatives, the percent identities, and the accession numbers of the sequences obtained are shown in Table 2, whereas Fig 1 shows the DGGE profiles. The raw original image of the DGGE gel is shown in S1 Fig.

**Table 2:** Sequencing results of the bands excised from the DGGE gel obtained from the amplified fragments of bacterial DNA extracted directly from the analyzed samples.

Samples	Bands^a^	Closest relative	% Ident.^b^	Acc. No.^c^
C	1	*Desulfomonile limimaris*	99	NR_025079
	2	*Agromyces cerinus*	97	NR_036873
	3	*Aliihoeflea* sp.	97	JN210898
HC	4	*Streptomyces* sp.	97	MK702081
	5	*Lysobacter rhizophilus*	97	NR_152715
	6	*Streptomyces* sp.	95	MK702081
	7	*Corynebacterium jeikeium*	97	NR_118863
	8	*Kytococcus sedentarius*	97	NR_074714
	9	*Roseomonas oryzae*	98	NR_137403
	10	*Deferribacter desulfuricans*	97	NR_075025
HCF	11	*Hymenobacter coalescens*	97	NR_151971
	12	*Massilia alkalitolerans*	97	NR_043094
	13	*Blastochloris viridis*	98	NR_041712
	14	*Blastochloris viridis*	97	NR_041712
ZHC	15	*Mobilicoccus pelagius*	98	NR_113143
	16	*Pseudonocardia xishanensis*	98	NR_108411
	17	*Saccharopolyspora* sp.	96	MH777902
	18	*Corynebacterium* sp.	97	MH400695
	19	*Phycicoccus endophyticus*	97	NR_148775
	20	*Saccharomonospora* sp.	97	KP639601
	21	*Corynebacterium halotolerans*	97	NR_102500
S	22	*Phycicoccus endophyticus*	97	NR_148775
	23	Failed	-	-
	24	*Saccharomonospora* sp	96	KP639601
HS	25	*Lysobacter* sp.	98	MG237861
	26	*Rhodocyclus purpureus*	97	NR_044679
	27	*Aliihoeflea* sp.	97	JN210898
	28	*Lysobacter* sp.	97	MG237861
HSF	29	*Pseudomonas* sp.	98	KX079778
	30	*Bacillus* sp.	97	KX681796
	31	*Phycicoccus endophyticus*	97	NR_148775
	32	*Janibacter* sp.	97	DQ268766
	33	*Microbacterium* sp.	98	JN935776
ZHS	34	*Shewanella* sp.	97	MF155923
	35	*Bacillus* sp.	98	KX785165
	36	*Lysobacter* sp.	97	MG237861
	37	*Phycicoccus endophyticus*	97	NR_148775
	38	*Shewanella* sp.	97	MF155923
	39	*Microbacterium sp*.	98	JN935776

^a^ Bands are numbered as indicated in Fig 1.

^b^ Percentage of identical nucleotides in the sequence obtained from the isolates and the sequence of the closest relative found in the GenBank database.

^c^ Accession number of the sequence of the closest relative found by BLAST search.

C, coffee by-product; HC, *H*. *illucens* reared on coffee by-product; HCF, frass of *H*. *illucens* reared on coffee by-product; S, vegetable substrate; HS, *H*. *illucens* reared on vegetable substrate; HSF, frass of *H*. *illucens* reared on vegetable substrate; ZHC, gut content of zebrafish fed HC; ZHS, gut content of zebrafish fed HS.

**Fig 2:**
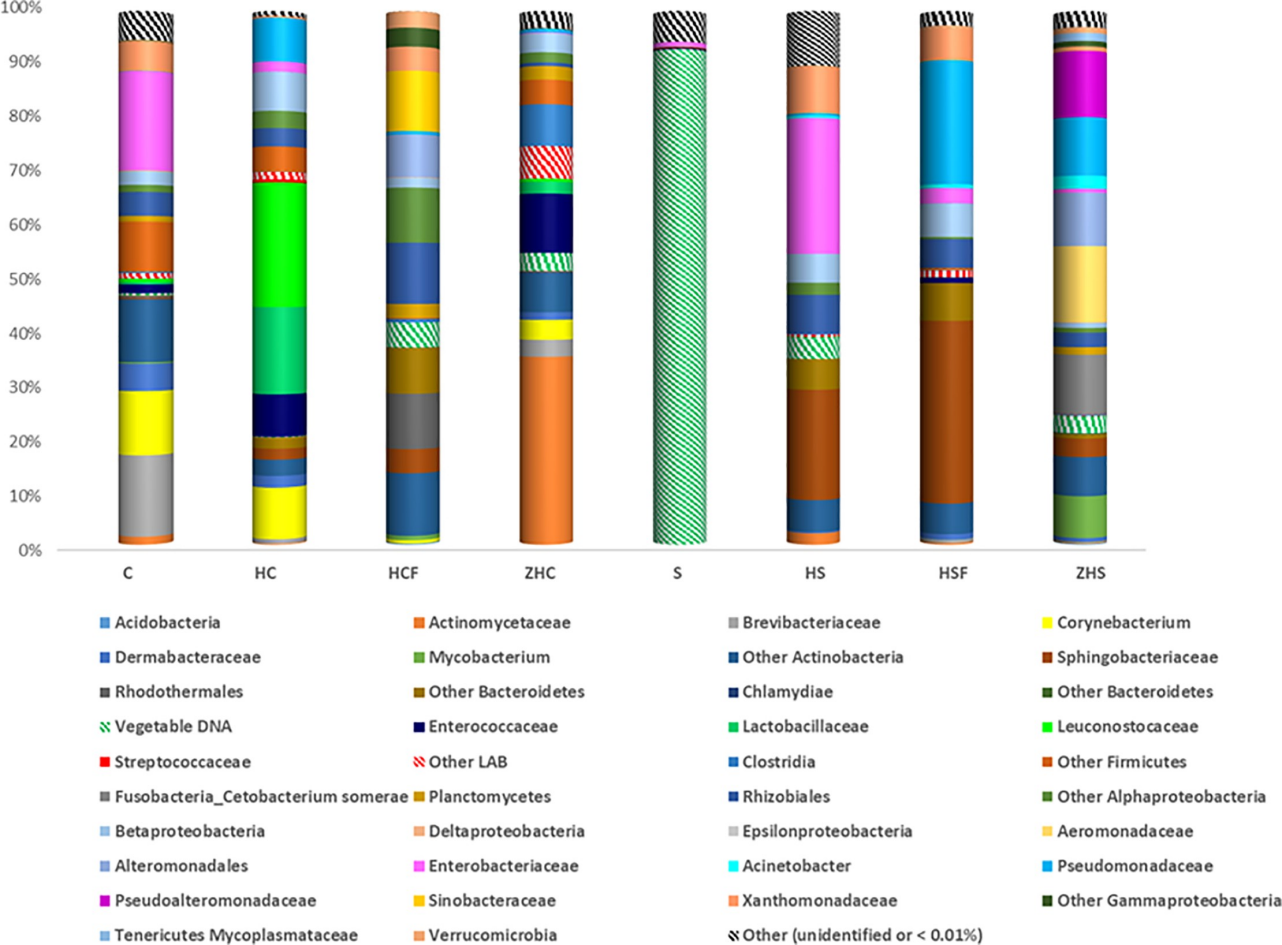
Relative abundances (%) of bacterial community in the analyzed samples identified by analysis of V3-V4 region of the 16S rRNA gene through MiSeq Illumina. C, coffee by-product; HC, *H*. *illucens* reared on coffee by-product; HCF, frass of *H*. *illucens* reared on coffee by-product; S, vegetable substrate; HS, *H*. *illucens* reared on vegetable substrate; HSF, frass of *H*. *illucens* reared on vegetable substrate; ZHC, gut content of zebrafish fed HC; ZHS, gut content of zebrafish fed HS.

Moreover, the results of Illumina sequencing are reported in Fig 2; in addition, raw values of relative abundance (% of bacterial community) in the analyzed samples identified by analysis of V3-V4 regions of the 16S rRNA gene through MiSeq Illumina are reported in S2 Table. In more detail, the DNA extracted from the samples successfully amplified the bacterial V3-V4 16S rRNA gene region. After splitting and quality trimming the raw data, 453,815 reads remained for subsequent analysis. After alignment, the remaining operational taxonomic units (OTUs) were clustered at a 3% distance. To analyze the bacterial community richness of each sample, the number of OTUs, the coverage estimator, the Shannon diversity index and the Chao1 richness estimator were determined using QIIME at 97% similarity levels (Table 3). Good's estimator of coverage was over 99% for all the samples, which indicated that most of the bacterial phylotypes were detected. Based on the Shannon and Chao indices, pooled samples HC, HS and HSF showed the highest richness and evenness of bacterial communities, while pooled samples C, ZHC and S exhibited the lowest.

**Table 3:** Number of sequences analyzed (N reads), diversity richness (Chao 1), observed operational taxonomic units (OTUs), estimated sample coverage for 16S (Coverage) and diversity index (Shannon) for samples from the two rearing cycles.

Sample	N reads	Chao 1	OTUs	Coverage	Shannon
C	60,235	447	417	99.95	8,059
HC	65,288	1,164	931	99.77	8.774
HCF	60,530	614	565	99,92	8.361
ZHC	44,728	347	346	99,97	7.587
S	50,489	460	442	99,95	7.090
HS	49,096	1,113	914	99,65	8.857
HSF	63,101	1,430	1,151	99,79	9.079
ZHS	60,348	856	761	99,82	8.322

C, coffee by-product; HC, *H*. *illucens* reared on coffee by-product; HCF, frass of *H*. *illucens* reared on coffee by-product; S, vegetable substrate; HS, *H*. *illucens* reared on vegetable substrate; HSF, frass of *H*. *illucens* reared on vegetable substrate; ZHC, gut content of zebrafish fed HC; ZHS, gut content of zebrafish fed HS.

It is notable that, only for pooled S samples, vegetable DNA and a few saprophytic microbial species were observed; these data are consistent with those recently reported by Osimani et al. [27] for wheat meal used as insect feed. Pooled C samples revealed the dominance of Enterobacteriaceae, Brevibacteriaceae, *Corynebacterium*, Xanthomonadaceae and Rhizobiales (within this latter family, *Aliihoeflea* sp. was also detected via PCR-DGGE). The latter two taxa showed elevated values of percent relative abundance in pooled HCF samples. Pooled HC samples were characterized by the dominance of Leuconostoccaceae, Lactobacillaceae and Enterococcaceae, which were minorities in C, thus suggesting a positive selective pressure for these taxa in the insect gut. As reported by Costa et al. [34], chlorogenic acid and caffeic acid represent important compounds in coffee by-products (such as silverskin) with potentially inhibitory effects on bacterial growth. Interestingly, different authors [35–37] have reported that such compounds may also stimulate the growth of lactic acid bacteria (e.g., *Lactobacillus collinoides*, *Lactobacillus brevis*, etc), thus likely explaining the massive presence of Lactobacillaceae or Leuconostoccaceae in HC samples. It is noteworthy that lactic acid bacteria have been widely detected in different edible insect species with potential symbiotic or probiotic activities [38].

Among Actinobacteria detected in HC, PCR-DGGE allowed the presence of *Streptomyces*, *Corynebacterium*, and *Kytococcus* to be discovered. It is noteworthy that among *Streptomyces*, the species *Streptomyces griseus* and *Streptomyces coelicolor* is a chitinolytic bacterial species that can improve chitin digestibility in insect-based feeds [39, 40]. *Corynebacterium* has very recently been found in the gut of *H*. *illucens*, with functions in hydrogen metabolism, nitrogen cycling, and sulfur compound metabolism [41]. *Kytococcus* comprises Gram positive species of environmental origin [42]; although found as minority species, to the authors’ knowledge this is the first report of *Kytococcus* in the microbiota of edible insects.

As already described for viable counts, reductions in Enterobacteriaceae relative abundance were observed in both HC and HCF. It is noteworthy that, regarding Enterobacteriaceae, HC was also characterized by lower abundance than in HS. It is likely that the presence of bioactive compounds from the coffee by-product, as chlorogenic or caffeic acids, could have modulated the growth of this bacterial family. Indeed, Zhang et al. [43] recently reported a decrease in the population of *Escherichia coli* in the colons of pigs fed diets supplemented with chlorogenic acid. Moreover, De Smet et al. [16] reported that the effectiveness of pathogen reduction by *H*. *illucens* can be affected by the composition of the substrate, thus supporting the results obtained in the present study.

Pooled HCF samples were also characterized by the presence of a rich microbiota mainly composed of Alteromonadales, Rhizobiales, Bacteroidetes, Rhodothermales, and Sphingobacteriaceae. To the authors’ knowledge the microbiota of *H*. *illucens* frass is actually poorly studied; hence, no further comparison is possible. It is noteworthy that, as reported by Mitchell and Hanks [44], frass can represent a vehicle for transmission of bacterial plant pathogens as well as a rich culturing media for saprophytic microorganisms. Moreover, the frass microbiota can synthesize chemical compounds that can be perceived by other insects, thus affecting the oviposition of some species [45]. Finally, insect frass used as fertilizer can also represent a source of safety risks for consumers due to the possible presence of human pathogens [46]; hence, a careful microbiological assessment of such a by-product must always be included in risk assessment.

In pooled HS samples the dominance of Enterobacteriaceae, Sphingobacteriaceae, Rhizobiales (among which closest relatives to *Aliihoeflea*) and Xanthomonadaceae (among which closest relatives to *Lysobacter*) was highlighted.

Sphingobacteriaceae have already been found in the microbiota of crambid butterflies, whereas the occurrence of species belonging to Xanthomonadaceae has already been reported in butterflies, beetles and crickets, thus confirming the association of these two bacterial families with the microbiota of insects [47, 48]. Finally, Rhizobiales have recently been detected in flea microbiota by Nziza et al. [49]. Members of Rhizobiales (e.g., *Bartonella*) can represent facultative intracellular parasites able to cause infection in human or wild and domestic animals [50].

In pooled HS samples, a low occurrence of Pseudomonadaceae was also observed; this bacterial family was in turn highly represented in the pooled HSF samples, together with Sphingobacteriaceae and Enterobacteriaceae.

The microbiota of insects can be strongly influenced by feeding substrates and vertical transmission from mother to offspring [27]. In the present study, the coffee by-product may have exerted a selective pressure on the microbiota of HC due to the presence of bioactive compounds such as chlorogenic acid and caffeine [51], thus perhaps explaining the differences in microbial composition between HC and HS. The results obtained in the present study are consistent with those very recently reported by Bruno et al. [15] which demonstrated that feed substrate can affect *H*. *illucens* midgut microbiota composition.

Zebrafish gut samples (ZHC and ZHS), obtained from the two feeding trials, were characterized by specific microbial patterns in which Actinobacteria and Alteromonadales were always detected, irrespective of the diet used.

Among Actinobacteria, PCR-DGGE allowed the presence of closest relatives to *Mobilicoccus*, *Pseudonocardia*, *Saccharopolyspora*, *Corynebacterium*, and *Phycicoccus* to be discovered in pooled ZHC samples. Moreover, among Alteromonadales, closest relatives to *Shewanella* were detected by PCR-DGGE in pooled ZHS samples, together with Actinobacteria genera (*Phycicoccus* and *Microbacterium*).

Actinobacteria have already been detected in the gut microbiota of zebrafish, thus constituting one of the major microbial taxa involved in the function of the intestinal barrier of the fish and playing an essential role in the synthesis of antibiotics against fish pathogens [52]. Hence, the detection of Actinobacteria in both ZHC and ZHS pooled samples suggests healthy status of their gut systems.

High occurrence of Alteromonadales has been associated with good fish health in recirculating aquaculture systems [53]. Moreover, the presence of *Shewanella* detected in the present study is consistent with observations of Rimoldi et al. [54], who described this bacterial genus as part of the core microbiota detected in the gut of rainbow trout (*Oncorhynchus mykiss*) fed *H*. *illucens*-based diet.

Interestingly, the presence of Enterobacteriaceae in fish guts was higher in ZHS samples than in ZHC, thus suggesting a different contribution of the two feeding substrates (HC or HS) in the modulation of Enterobacteriaceae in the fish gut. Moreover, fish fed HC were characterized by the abundant presence of Actinomycetaceae, which, as recently reported by Nurul et al. [55], can establish a symbiotic association in fish with benefits for the bacteria and the host.

Finally, a higher relative abundance of Clostridia was detected in ZHC than in ZHS. This finding could likely be explained by the stimulating activity of chlorogenic acid and caffeine, contained in the zebrafish diet (HC), on this bacterial taxon [56].

## Conclusions

Overall, the data collected suggest that the microbiota of H. *illucens* may have been influenced by the feeding substrates. Similarly, zebrafish gut microbiota may have been influenced: i) by the microbiota of *H*. *illucens*-based feed and ii) eventually by insect-derived bioactive compounds contained in the experimental diets. Indeed, as recently reported by Bruni et al. [57], the fish gut microbial community is plastic and can be influenced by insect-based feed. Moreover, the results of both PCR-DGGE and Illumina sequencing showed the presence of a core microbiota constantly associated with the zebrafish gut, irrespective of the diet.

As highlighted by Huyben et al. [58], a high bacterial diversity can lead to improved health fish guts. Indeed, a rich microbial community could increase host resistance to pathogen invasion and intestinal infection. Hence, the low microbial richness exhibited by ZHC should be carefully considered when diets including HC are suggested.

Further research is needed to understand the interactions among *H*. *illucens* microbiota, *H*. *illucens* bioactive compounds (i.e., chitin, polyphenols, etc.) and the zebrafish gut microbiota.

Moreover, data collected on insect frass can be useful to widen the knowledge on such a poorly investigated insect rearing by-product for its further use.

## Compliance with ethical standards

All procedures involving animals were conducted in compliance with Italian legislation on experimental animals and were approved by the Ethics Committee of the Università Politecnica delle Marche (Ancona, Italy) (84/94-A). All efforts were made to minimize animal suffering by using an anesthetic (MS222; Sigma Aldrich, Saint Louis, Missouri, USA).

## Supporting information

S1 Table Raw data used for statistical analysis of viable counts. (XLSX)

S2 Table Raw data of relative abundances (%) of bacterial community in the analyzed samples identified by analysis of V3-V4 region of the 16S rRNA gene by using MiSeq Illumina. (XLSX)

S1 Fig Raw original image of bacterial DGGE profiles of the DNA extracted directly from the analyzed samples and amplified with primers 338fGC and 518r. (PDF)
