# Supplementary material for: Hermetia illucens in diets for zebrafish (Danio rerio): A study of bacterial diversity by using PCR-DGGE and metagenomic sequencing
Source: PLoS One. 2019 Dec 10;14(12):e0225956. doi: 10.1371/journal.pone.0225956 (PMC6903733; doi:10.1371/journal.pone.0225956)

S1 Fig. Raw original image of bacterial DGGE profiles of the DNA extracted directly from the analysed samples and amplified with primers 338fGC and 518r

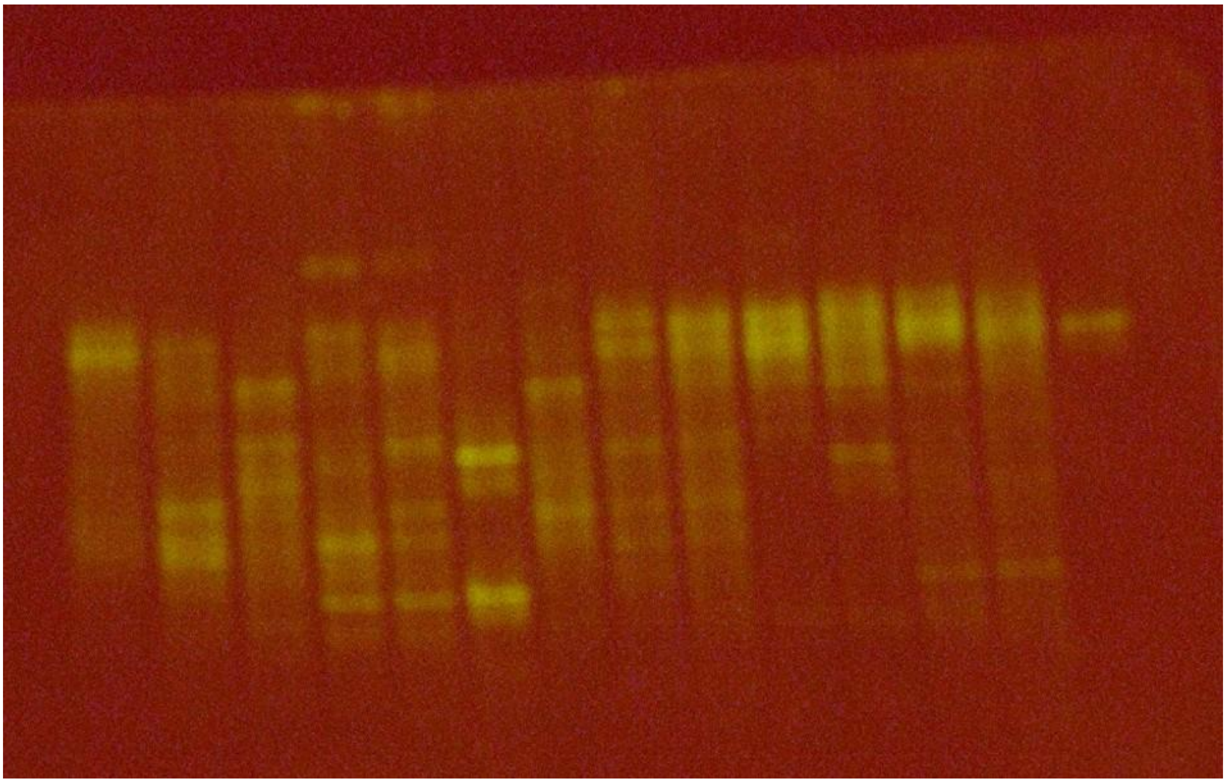

Supplement: S1 Fig — (PDF) [file pone.0225956.s003.pdf]
